# The Relationship between Typical Environmental Endocrine Disruptors and Kidney Disease

**DOI:** 10.3390/toxics11010032

**Published:** 2022-12-29

**Authors:** Xing Zhang, Jodi A. Flaws, Michael J. Spinella, Joseph Irudayaraj

**Affiliations:** 1 Department of Bioengineering, University of Illinois Urbana-Champaign, Urbana, IL 61801, USA; 2 Department of Comparative Biosciences, College of Veterinary Medicine, University of Illinois Urbana-Champaign, Urbana, IL 61801, USA; 3 Carl Woese Institute for Genomic Biology, University of Illinois Urbana-Champaign, Urbana, IL 61801, USA; 4 Cancer Center at Illinois, University of Illinois Urbana-Champaign, Urbana, IL 61801, USA; 5 Beckman Institute of Technology, University of Illinois Urbana-Champaign, Urbana, IL 61801, USA

**Keywords:** endocrine disrupting chemicals, kidney diseases, renal cell carcinoma, disease mechanisms

## Abstract

Endocrine disrupting chemicals (EDCs) are exogenous substances that alter the endocrine function of an organism, to result in adverse effects on growth and development, metabolism, and reproductive function. The kidney is one of the most important organs in the urinary system and an accumulation point. Studies have shown that EDCs can cause proteinuria, affect glomeruli and renal tubules, and even lead to diabetes and renal fibrosis in animal and human studies. In this review, we discuss renal accumulation of select EDCs such as dioxins, per- and polyfluoroalkyl substances (PFAS), bisphenol A (BPA), and phthalates, and delineate how exposures to such EDCs cause renal lesions and diseases, including cancer. The regulation of typical EDCs with specific target genes and the activation of related pathways are summarized.

## 1. Introduction

Endocrine disrupting chemicals (EDCs) are a class of hormone-like chemicals that exist in the environment and interfere with the production, transport, metabolism, regulation, degradation, and/or action of hormones. The ability of EDCs to interfere with endogenous hormones can lead to adverse effects on development, reproduction, immune, endocrine, and nervous systems of the organism. The cascade of events can also result in endocrine and metabolic imbalances in offspring as well [1,2]. EDCs are present in wastewater, textiles, cosmetics, waste residues produced by industrial and agricultural processes and in several domestic and household goods [3,4,5], and ultimately end up in landfills. Common contaminants include per- and polyfluoroalkyl substances (PFAS), polychlorinated biphenyls (PCBs), polycyclic aromatic hydrocarbons (PAHs), dioxins, bisphenol A (BPA), and phthalates, as well as heavy metals such as Cd, Pb, Hg [6]. Upon entry into the human system, EDCs often indirectly interact to cause endocrine imbalance in the organism by affecting the formation, secretion, transport, and metabolism of hormones in the organism, and thus, affecting growth, development, and reproduction.

**Figure 1:**
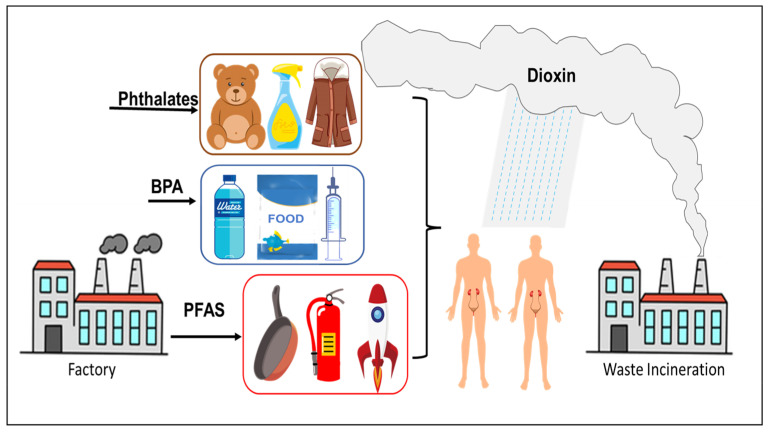
Common sources of EDCs.

Due to the environmental persistence of EDCs and their mobility in water bodies, these chemicals can migrate for a long distance along with water bodies [7,8,9]. At present, EDCs such as PFAS, BPA, PCBs and PAHs have been found in main water bodies worldwide [10,11]. Due to the limitations in existing wastewater treatment technologies, EDCs from livestock and poultry as well as domestic and industrial wastewater enter natural water bodies with wastewater discharge and continue to migrate and transform. EDCs in the environment can also be enriched through the food chain and accumulate in organisms at all trophic levels. The higher the trophic level, the higher the accumulation, eventually leading to toxicity [12,13,14]. There are several standard methods for extracting and estimating the concentration of EDCs in biological and environmental samples, including solid-phase extraction (SPE) [15], liquid chromatography-mass spectrometry (LC-MS) [16], gas chromatography-mass spectrometry (GC-MS) [17,18] and high-performance liquid chromatography (HPLC) [15,19]. It is important to use appropriate sample extraction and estimation methods for accurate and reliable detection of the levels of EDCs in different types of samples. With extensive advances in the chemical industry and the ubiquitous presence of plastics, EDCs entering the environment are on the rise both in terms of quantity and variety (new materials), and consequently, their hazards to humans and animals have attracted increased attention by societal and regulatory agencies (Figure 1).

The kidney is one of the main target organs of EDCs for accumulation. Patients with kidney disease usually show decreased renal function and proteinuria, which affects recovery from the disease [20]. Common renal diseases include chronic nephritis, renal calculi, renal failure, and renal cysts. These diseases are usually associated with the glomerular filtration rate (GFR) of the kidney, pathological damage, and abnormal blood or urine composition [21,22].

EDCs have attracted considerable public, regulatory, and scientific attention given their impact on kidney diseases and kidney cancer. Despite past efforts, less is known on mechanisms of action of the different EDCs on kidney function. In this review, we focus on the toxicity of non-metallic and non-agricultural EDCs that are used as additives (plasticizers phthalates and bisphenol A) and industrial chemicals (polychlorinated biphenyls, dioxins, PFAS, flame retardants) in triggering major renal diseases including cancer associated with exposures (Table 1).

## 2. Dioxin

**Figure 2:**
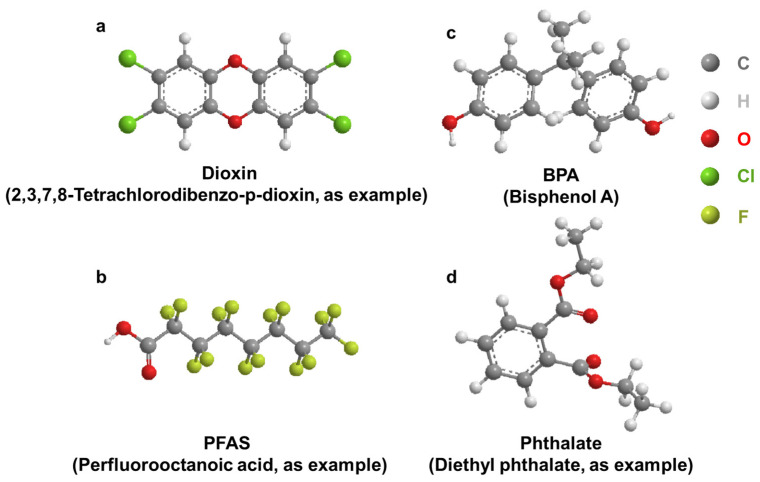
General structure of select EDCs. (**a**): Dioxin; (**b**): BPA; (**c**): PFAS; (**d**): Phthalate.

Dioxins were identified as toxic compounds in the 1960s. Dioxins are a group of structurally related chemicals composed of two coplanar benzene rings (Figure 2a). These compounds induce a similar spectrum of toxic phenotypes, with a wide range of potency. 2,3,7,8-tetrachlorodibenzo-*p*-dioxin (TCDD) is one of the most toxic compounds in this group of chemicals [23]. Incineration is the largest source of dioxin emissions in the environment [24,25]. Dioxins enter the ambient air through chimneys, spread continuously, and accumulate in the surrounding area of the incineration plant. Routes of exposure to dioxins include inhalation, dust ingestion, and skin contact. Dioxins accumulate in the tissues of various animals [26,27,28,29,30,31] and cause chloracne, embryotoxicity [32,33] and nephrotoxicity [34].

### 2.1. Accumulation of Dioxins in the Kidney

*Animal studies*: Several studies, but not all, suggest that TCDD may accumulate in the kidney to impart toxicity. Examination of aquatic organisms by analyzing ten-year-old fish in heavily polluted lakes in China showed that a large amount of dioxin accumulated in the kidneys [35]. Numerous studies have indicated that the accumulation of TCDD can cause the ballooning degeneration or even necrosis of the renal tubules of zebrafish [36]. Polybrominated diphenyl ethers (PBDEs), which have similar structures to PCB, have also been associated with renal histopathological changes [37] and significantly reduced catalase activity [38]. Exposure of infant mice to PCB caused hyperuricemia in adults, leading to secondary nephrotoxicity such as renal hypertrophy and fibrosis [39]. After 12 days of exposure, the combined exposure to TCDD and PCB was more likely to induce nephrotoxicity through high expression of *CYP1A1* (Cytochrome P450 Family 1 Subfamily A Member 1), compared with the control group (equivalent volume of olive oil). TCDD and PCB exposure also significantly increased serum creatinine and blood urea nitrogen levels, renal oxidative stress and histopathological changes compared to control in rats [40]. Further studies will shed light on TCDD and PCDD mediated carcinogenesis [41].

*Human studies*: Shalat et al. [42] reported that three young male utility workers developed kidney cancer after chronic exposure to PCB-containing transformers. Residents from an e-waste dismantling area showed increased accumulations of PCB, which could have contributed to abnormal changes in markers of kidney injury [43]. The screening of environmental chemicals in the soil of an e-waste recycling area and human cancer risk assessment calculations showed that dioxins have the highest potential cancer risk to residents, followed by PCBs [44]. Through a multiple linear regression model analysis of 150 pregnant women, it was found that exposure to environmental pollutants may have negative effects, while exposure to greenspace may have positive effects on fetal renal function during pregnancy [45]. Epidemiological evidence indicated that the development of diabetes and chronic kidney disease was also associated with long-term exposure and accumulation of dioxins [46,47,48]. Jain [48] analyzed data from US adults from 1999 to 2004 to investigate concentration changes of four dioxin homologs and four separate furan homologs at various stages of renal function decline and found that renal dysfunction was associated with high dioxin/furan concentrations.

### 2.2. Effect of Dioxin on AHR Regulation/Activity and RCC

The **AHR** is a ligand activated transcription factor that mediates the toxic effects of TCDD. The **AHR** is mostly expressed in the nucleus of advanced clear cell renal cell carcinoma (RCC) and tumor infiltrating lymphocytes, and its expression is related to the stage and histological grade of pathological tumors [49]. Numerous studies have shown a complex association between the **AHR** and cancer characteristics, including increased malignant cell invasion, migration, metastasis, and survival [50,51,52]. The primary structure of the **AHR** is considered to be critical to determining the sensitivity and specificity of animal responses to dioxins.

*Animal studies*: In a constructed adenine diet model of chronic kidney disease, female **AHR** knockout mice showed inflammatory and pro-fibrotic gene expression and acute tubular injury [53]. After exposure to TCDD, 9 renal function was significantly reduced in wild-type male mice, indicating that the **AHR** plays a major role in mouse kidney development [54]. Due to poor renal excretion in patients with chronic kidney disease, the accumulation of toxic substances was found to increase *CYP1A1* expression. Because of the higher inducibility of polymorphic genotypes, the pathway may become more deleterious in individuals with homozygous mutant alleles [55].

TCDD-induced fetal hydronephrosis (TiFH) is a type of obstructive hydronephrosis characterized by the presence of dilated ureter or ureteral effusion. The relationship between TiFH and **AHR** was investigated in both rats and mice. In mice, *Cox-2* (cyclooxygenase-2) plays a key role in TiFH [56,57]. The induction effect of TCDD in the mouse kidney does not require translocation of **AHR** to the nucleus. TCDD induction of *Cox-2* in mouse kidney is primarily mediated by a non-genomic pathway that activates **AHR**. In rats, TiFH is also induced and may be an endogenous ligand for **AHR** and/or a protein interacting with **AHR**. In contrast to rats, mice lacking **AHR** did not develop hydronephrosis or hydronephrosis in the absence of TCDD [58]. To better explore the role of **AHR** in normal development and chemical response, **AHR** knockout (**AHR**-KO) models were created in rats and mice, respectively. However, in **AHR**-KO rats, hydronephrosis and hydroureter were observed and **AHR** was found to play significantly different roles in tissue development and virulence in rodent species [59].

*Human studies*: In a study of more than 300 chronic kidney disease (CKD) patients and healthy controls, overexpression of polymorphic variants of *CYP1A1* were associated with free radical production related enzymes after exposure to environmental pollutants, and with induction of renal dysfunction. Because of the different effects of **AHR** in rats and mice, it was not possible to directly use animal models to verify the effect of TCDD in the human kidney. To better detect the effects of dioxins on human health and reduce the differences between species, the mouse **AHR** was replaced with human **AHR** cDNA by knock-in strategy. Human **AHR** can be expressed in mice to mediate the development of TCDD-induced hydronephrosis [60]. Transcriptional analysis of human **AHR** was performed and compared in the liver and kidney, but dioxin exposure in the kidney altered only 17 genes, including many **AHR** target genes [61].

Overall, the relationship between *CYP1A1*, TCDD, and the **AHR** is complex and involves the metabolism of TCDD by *CYP1A1* and the activation of the **AHR** by TCDD. The activation of the **AHR** by TCDD may lead to the expression of various genes that could contribute to nephrotoxicity, including genes involved in inflammation and oxidative stress. *CYP1A1* is involved in the metabolism of TCDD, and the activation of the **AHR** by TCDD may also lead to the expression of *CYP1A1*. More research is needed to fully understand the mechanisms by which TCDD causes adverse health effects.

## 3. Per- and Polyfluoroalkyl Substances

Per- and polyfluoroalkyl substances are a class of chemicals (Figure 2b) used in many industrial and consumer products with the main function of resisting heat, stains, water, and grease [62,63]. Classic examples include Teflon, coating on fast-food wrappers, non-stick pans, floor polish, carpets, furniture fabrics, firefighting foams, clothing treatments, and many others [64,65]. It has been estimated that over 98% of Americans have these chemicals in their body [66,67,68,69]. PFAS can be classified into long chain and short chain chemicals based on the number of carbon atoms (usually between 4 and 12) and they have a long half-life and are difficult to metabolize (because of the strong covalent C–F bond). In general, shorter chain chemicals (n ≤ 6) are easier to excrete than longer chain chemicals (n ≥ 6). Previous studies have found that urine was the main mode of excretion of PFAS in mice and monkeys [70,71]. High concentrations of PFAS were detected in human urine, and renal excretion in humans and accounts for approximately one-fifth of the total excretion based on their serum half-lives [72,73]. Analysis of blood and urine samples from adults showed that a variety of PFAS were detected, including perfluorooctane sulfonates (PFOS), perfluorooctanoic acid (PFOA), perfluorononanoate (PFNA), perfluorohexanesulfonic acid (PFHxS), and perfluorinated decanoic acid (PFDA), and that the level of PFAS in urine was positively correlated with that in blood [74].

### 3.1. Accumulation of PFAS in the Kidney

Renal excretion is an important excretory pathway of PFAS in humans and animals, and the kidney plays an essential role in the metabolism and transport of PFAS. After exposure to PFAS in rainbow trout, the degree of accumulation was blood > kidney > liver > gallbladder [75,76]. The distribution of 24 types of PFAS in eight different tissues of *Orcinus orca* was reported, and the highest concentration of PFAS was found in the liver, followed by the blood and kidney [77]. PFAS were measured in samples from 31 harbor porpoises (*Phocoena relicta*) stranded on the Black Sea coast and PFOS was found to account for 90% of all PFAS, which were highest in the liver (327 ± 351 ng/g wet weight) and kidney (147 ± 262 ng/g wet weight) tissues [78]. Both long-chain and short-chain PFAS were also detected in the organs and tissues of the sea leopard (*Phoca vitulina*) in the Netherlands [79,80]. In another study, PFAS accumulation in the tissues and organs of the Baltic guillemots (*Uria algge*) were noted. PFOS remained the most abundant, with a median concentration of 127 ng/g weight in the kidney [81]. The distribution of 16 PFAS in the liver, blood, kidney, adipose tissue, and muscle of 18 Arctic foxes aged 1–3 years was also noted, with the concentration of PFAS being highest in the liver, followed by the blood and kidney [82]. These data suggest that the tissue distribution and accumulation patterns of PFAS in organisms vary considerably. However, the kidney has always been an important organ for PFAS accumulation in animal species. Compared with long-chain PFAS, short-chain PFAS (e.g., perfluorobutyric acid, PFBA) have a relatively short half-life in blood and do not accumulate at high levels in human kidney tissue based on the analysis of seven lung and nine kidney samples from cancer patients [83]. Since studies on kidney toxicology are sparse, this makes a strong argument for investigations on chronic exposure in relation to accumulation and kidney diseases.

### 3.2. Relationship between PFAS and CKD

PFAS are ubiquitous and difficult to metabolize, and thus, their possible toxic effects on animals and humans have attracted significant attention [84,85,86,87,88,89,90]. However, some studies suggest that there may be no causal relationship between chronic kidney disease (CKD) and PFAS. To investigate the longitudinal association between PFOA and PFOS exposure and the incidence of CKD in patients with diabetes, PFOA and PFOS levels in serum were measured in nearly 1000 patients with diabetes and it was found that PFOS levels were significantly associated with a lower risk of CKD incidence. The interaction between PFOA and PFOS exposure and CKD was not statistically significant [91]. In a study of 73 normal weight pregnant women who did not have gestational diabetes mellitus or preeclampsia, the concentration of PFNA, PFOA, PFOS, and PFHxS in the blood changed from early to late pregnancy. The levels of these PFAS decreased during pregnancy, but were not linked to changes in the estimated glomerular filtration rate (eGFR) or glomerular pore size. Collectively, these studies suggest that changes in renal function are not caused by PFAS [92].

On the contrary, other studies have shown that PFAS was closely related to a decline in renal function. EGFR was one of the diagnostic factors of CKD, and studies have shown that PFAS can affect the level of eGFR. PFAS levels in 61 CKD patients were found to be significantly higher than in the healthy control group [93]. The levels of hemoglobin, serum albumin, and eGFR were significantly lower while the levels of potassium and uric acid were higher in the CKD group. PFOS levels were found to be significantly higher in CKD patients than controls. By measuring serum concentrations of PFOA, PFOS, PFNA, and PFHxS in adolescents living near chemical plants and comparing their eGFR with another 10,000 adolescents, the association between eGFR and serum PFOA might serve as an indicator of decreased renal function [94]. Serum samples from 53,650 adults (5210 with diabetes) were evaluated for four different PFAS. All PFAS had a strong positive correlation with eGFR in patients with CKD or anemia, and these relationships were more significantly associated in patients with diabetes. PFAS are inversely associated with renal function in CKD and diabetes, suggesting that exposure to PFAS could be associated with decreased renal function [95,96,97,98]. After following 1237 non-diabetic women aged 45 to 56 years until 2017, a study by Park [99] suggested that PFAS may increase the risk of diabetes in middle-aged women.

### 3.3. Effect of PFAS on RCC and Renal Function

Several discrepancies exist in studies on the association between PFAS and kidney cancer. Reports from 18 epidemiological studies on the association between PFOS exposure and human cancer risk indicated that the relative risks of PFOA and PFOS do not indicate a dose–response relationship between exposure and kidney cancer. Positive associations with PFOA exposure were detected in community settings, but because occupational exposure to PFOS was one to two orders of magnitude higher than environmental exposure, epidemiologic evidence does not support the hypothesis of a causal dose–response relationship between PFOS exposure and human cancer [100].

In contrast, other studies have demonstrated that the kidney was one of the target organs of PFAS. Data from 324 patients showed that a doubling of serum PFOA concentration was associated with an approximately 70% increase in RCC risk when PFAS concentrations were serially simulated. Higher concentrations of certain PFAS and the highest incidence of RCC were also observed in African Americans compared to non-Hispanic whites [101,102]. This study was also supported by the study from Steenland et al. [103], in which the association between PFOA and RCC was examined in nearly 1000 renal cancer cases. The pooled analysis indicated that PFOA was associated with renal cancer in human studies, for every 1 ng/mL increase of serum PFOA, the logarithmic ratio of RCC increased by 0.1349. These results provide strong evidence that PFOA is a renal carcinogen.

Since the association between PFOA and RCC has yielded conflicting conclusions in different studies, PFOA exposure was converted to serum PFOA concentrations and meta-analysis was used to estimate cancer risk. In the meta-analysis, risk for RCC rose 16% for each 10 ng/mL increase in serum PFOA. Thus, the association of PFOA with RCC is most likely causal and makes a strong argument for further research on the mechanisms underlying this association [104].

Recently, studies are beginning to emerge on the effect of PFAS in select cancers (liver, prostate, kidney, etc.) [105], but mechanistic studies on the role of PFAS are sparse. Although epidemiological evidence points to kidney cancer as one of the cancers triggered by PFAS, very few studies have examined the role of PFAS in kidney disease or renal cell carcinoma.

A study by Park [106] showed that after being absorbed by the human body, PFAS are first combined with serum proteins and then deposited in various organs of the body such as the liver, kidney, and testis, and that PFAS cannot be excreted, resulting in a variety of toxic outcomes. The effect of PFAS on gap junctional intercellular communication (GJIC) in a dolphin renal epithelial cell line showed that PFOS, perfluorooctane sulfonamide (PFOSA) and perfluorohexane sulfonic acid (PFHA) could rapidly inhibit GJIC, while perfluorobutane sulfonic acid (PFBS) had no significant effect on GJIC. In humans, urinary excretion of short-chain PFAS was found to be higher, whereas other elimination pathways were found to be more dominant for long-chain chemicals [107,108]. These results suggest that the toxic effects of PFAS on organisms could possibly depend on its structure rather than the properties of its functional groups.

Only a few studies exist on the mechanism of PFAS exposure and kidney response. Examination of over 70 studies studies in the fields of epidemiology, pharmacokinetics, and toxicology showed that PFAS exposure causes renal tubular tissue and cellular changes. PFAS exposure altered several pathways associated with kidney disease, including oxidative stress, peroxisome proliferator-activated receptor (PPAR) pathway, and epithelial–mesenchymal transition (EMT) [109]. By evaluating the role of PFAS in Xenopus A6 renal epithelial cells, the inductive effects of these chemicals were attributed to the stimulation of DNA/RNA, secondary protein structures, lipids, and fatty acids, which ultimately lead to cell death [110]. PFOA exposed to male and female rats showed accumulation in liver, kidney, and small intestinal microsomes. Significant uridine diphosphate -glucuronosyltransferase (UDPGT) activity was observed in all tests, but no evidence of PFOA-glucuronide formation was observed either by highly sensitive radio-chromatography or by liquid chromatography-mass spectrometry (LC/MS) [111]. Recent studies by Wen et al. [112] showed that exposure to PFOS resulted in increased expression of renal injury markers *Acta2* and *Bcl2l1*, decreased DNA methylation, and the upregulation of histone demethylases *Kdm1a* and *Kdm4c*. In addition, PFOS was shown to induce renal injury and upregulation of transcription factors *Nef2l2*, *Hes1*, *Ppara*, and *Ppard*. These results suggest a potential adverse effect of PFOS on renal fibrosis and carcinogenesis.

A number of studies have shown that the kidney may be at risk due to exposure to EDCs, including PFAS. However, there are still several important knowledge gaps in our understanding of kidney disease and kidney cancer due to the overlapping populations, limited modeling boundaries, and lack of mechanistic studies. Elucidating the effects of PFAS exposure on genetic, biological, environmental, occupational, and other risk factors is critical. Further studies on the direct and indirect mechanisms of nephrotoxicity, including experimental models, metabolic analysis, and translation to epidemiology is warranted. In addition, how environmental toxicants such as PFAS drive differences in kidney disease among different populations remains poorly understood.

Occupational workers are commonly exposed to high levels of PFAS with greater risk compared to the general population. A study (data from 1950–2009) analyzing cancers and cancer deaths among ~30,000 firefighters (21–29 years of employment) found that cancers of respiratory, digestive, and urinary systems were the highest [113]. In a study of 40 occupational workers and 52 population-based controls in China, a total of 14 biomarkers related to oxidative stress, fatty acid β-oxidation disorder and kidney injury were identified in occupational workers [114]. HEK-293 cells were exposed to three widely used aqueous film forming foams (AFFFs). All AFFFs induced cytotoxicity and markedly inhibited cell proliferation when exposed to only one-tenth of the working concentration of fire suppression [115]. Therefore, the health effects of occupational exposure to PFAS on workers should not be ignored [116]. Studies from meta-analysis focused on cancer risk among firefighters indicated that firefighters were at increased risk of developing multiple myeloma, non-Hodgkin lymphoma, prostate, kidney, lung, and testicular cancer. Eight additional cancers were also listed as having a “possible” association with exposures to fire training activities [117,118]. Risk for these cancers among firefighters may be related to direct exposures to complex toxicants during fire training activities through inhalation and contact with PPE contributing to the modulation of biochemical or physiological pathways that put firefighters at increased baseline risk of developing cancer [119]. Cancer is also one of the leading causes of death among firefighters according to the Centers for Disease Control and Prevention [113,117,118]. Research is needed to reveal toxicity due to occupational exposure in the study of PFAS.

Currently, many traditional PFAS are being phased out and are being replaced by commercial compounds such as GenX, F-53B, and short-chain variants. Even more challenging is the existence of hundreds of undiscovered PFAS compounds whose health effects are unknown. Because of the dramatic increase in the production of novel alternative PFAS compounds, there is an urgent need to understand the relationship between PFAS exposure and kidney disease to find key biomarkers triggered due to PFAS toxicity.

## 4. Bisphenol A

Bisphenol A is an environmental toxicant with a phenolic ring and structural similarity to phenols (Figure 2c). It is an endocrine disrupter involved in the synthesis of a variety of plastics and it has been used since the 1960s to make the inner coating of plastic bottles, food packaging, and medical devices [120].

### 4.1. Accumulation of BPA in the Kidney

BPA can be detected in virtually 100% of human urine samples [121,122,123]. Pharmacokinetic and biomonitoring data indicate that BPA is rapidly and efficiently metabolized after ingestion [124]. Low doses of BPA are effectively reduced in the gastrointestinal tract and liver of adults of all species, with final exposures typically below 1% of the total. However, it is worth noting that, compared with neonatal primates, newborn rodents are immature and do not have the ability to metabolize and excrete BPA, leading to BPA accumulation in serum or tissues, including fetal tissues [125,126].

A population survey in China from 2003 to 2006 showed that urinary excretion of BPA decreased with decreasing renal function. A correlation was shown between urinary excretion of BPA and albuminuria and the association of BPA with renal injury [127]. The decline of renal function was found to be not conducive to the elimination of BPA, leading to its accumulation, forming a vicious cycle [128,129,130].

### 4.2. Effect of BPA on Renal Function

BPA is cytotoxic and mutagenic in aquatic organisms, animals, and humans. BPA exposure is known to cause various adverse effects on the immune, endocrine, reproductive, developmental and nervous systems [131,132].

*Animal studies*: Histopathological evaluation of rats exposed to BPA for 30 days showed infiltrative and dilatative changes in renal tissues, leading to renal failure [133,134,135]. Significant increases in sodium, potassium and calcium concentrations were observed in response to BPA, indicating that BPA disrupts electrolyte balance, leading to renal dysfunction [136,137]. Fetal glomerular abnormalities and decreased glomerular formation were observed after being exposed to BPA during pregnancy [138]. Histological examination of the kidneys of BPA-exposed fish revealed glomerular atrophy and distortion, uriniferous tubule edema, necrosis and atrophy, severe hyperemia, and blood hemolysis [139].

*Human studies*: Substantial evidence shows that high levels of BPA in the blood are associated with kidney disease. Despite the inconsistent urinary BPA concentrations observed in patients with kidney disease, the statistical association with eGFR supports the association between BPA and glomerular filtration, suggesting BPA as a possible environmental factor that induces kidney injury [140]. Low doses of BPA can cause cytotoxicity in renal mouse podocytes, and studies in human kidney cells have also shown that BPA promotes kidney injury [141,142,143]. In a study of patients with CKD, an increase in serum BPA was found to be accompanied by a decrease in eGFR and renal function, potentially due to BPA accumulation [144,145,146]. A positive correlation between urinary BPA levels and serum uric acid levels in children [147] was noted. Interestingly, urinary BPA levels were lower in children with CKD than in healthy people. BPA exposure had no effect on renal function in children with CKD, possibly because pediatric patients with CKD may have healthier eating habits than the general population [148].

### 4.3. Mechanisms of BPA Kidney Disease Promotion

To better inform on the biomarkers of renal disease, it is important to understand the molecular mechanism of BPA-induced nephrotoxicity and carcinogenicity [149]. In vitro experiments showed that BPA reduced superoxide dismutase (SOD) activity and glutathione (GSH) levels, while promoting cellular apoptosis, reactive oxygen species (ROS) production, and DNA damage [150,151,152]. A study in rats showed that proteinuria and glomerular damage were associated with increased lipid peroxidation and decreased levels of the antioxidants, glutathione and superoxide dismutase. BPA is known to directly act on kidney mitochondria to cause oxidative stress and mitochondrial dysfunction, resulting in subsequent damage to the whole organ [153,154,155,156]. Longer exposure times involve transcriptional responses of immune-related genes, potentially due to BPA-related oxidative stress in inducing inflammatory responses in macrophages [157,158,159]. Evaluation of epigenetic toxicity has shown that BPA can lead to DNA methylation. Long interspersed transposable element-1 (*Line1*) and CCGG global methylation rates in fetal kidney were 77.9% and 77.0%, respectively [160]. Changes in the methylation of CpG promoter were detected in the *Rassf1a* and C-myc genes in cells [161].

BPA was found to activate autophagy and apoptosis-related signaling pathways. BPA exposure activated autophagy-related proteins (**ATG5**, **ATG7** and **MAP1LC3B/LC3B**) and decreased the expression of **NRF-2** and **HO-1** proteins [162,163,164,165]. BPA also activated the **AMPK/mTOR** (The 5′-adenosine monophosphate-activated protein kinase and mammalian target of rapamycin) signaling pathway, which triggers *P62/Lc3/Beclin1* signaling, leading to the formation of autophagosomes and autolysosomes, and ultimately, stimulating autophagy in renal cells [166,167]. BPA exposure down-regulated the expression of **PI3K** (phosphatidylinositide 3-kinases) and **AKT** (protein kinase B) and activated the *Bcl/Bax-Caspase 9-Caspase 3* (B-cell lymphoma/BCL2-Associated X-Caspase 9-Caspase 3) signaling pathways, leading to apoptosis and necrosis of renal cells [168]. Recent studies have shown that BPA-induced renal dysfunction was associated with ferriosis, which depends on ferritin phagocytosis, through the activation of **AMPK-mTOR-ULK1** (Unc-51-like kinase 1) axis [169].

Further studies are needed to assess the molecular mechanisms of BPA-induced nephrotoxicity. In addition, some studies interestingly suggest that when BPA is co-exposed with other environmental pollutants, compensatory mechanisms exist that can reverse the damage caused by each toxic substance on its own. Additional research is needed to better understand the underlying synergistic mechanisms [170].

## 5. Phthalates

Phthalates are planar aromatic hydrocarbons with side chains (Figure 2d). They are often used as universal plasticizers for polyvinyl chloride (PVC) [3]. Phthalates also are widely used in many consumer products and medical devices, resulting in a significant burden on human health [171,172,173,174,175,176]. They can accumulate in the kidney and cause renal dysfunction and decline [177,178,179,180].

### 5.1. Accumulation of Phthalates in the Kidney

Similar to BPA, phthalates are rapidly metabolized and absorbed in the gastrointestinal tract and eventually excreted in urine. When ingested orally, phthalates are mainly found in the liver and kidneys, but do not accumulate as much in other organs or tissues [181,182,183,184]. However, phthalates are almost always detected in animals due to their continuous exposure.

### 5.2. Effect of Phthalates on Renal Function

*Animal studies*: Several studies suggest that phthalates only affect kidney function in rodents, and that primate kidneys are not target organs. Previous research has shown that oral administration of a high dose of phthalates (500 mg/kg/day) affects the liver and kidney in rodents, but not in primates [185,186]. Phthalates can induce the epithelial–mesenchymal transition in rat renal cells and aggravate renal fibrosis [187]. Exposure to phthalates in the μg/liter range in drinking water induces significant perturbation of metabolic profiles and renal dysfunction in mice [188]. To assess the effects of phthalates in primates, young adult male cynomolgus monkeys were exposed to high doses (500 mg/kg/day) of phthalates via endogastric cannulators for 14 consecutive days. Histopathological examination revealed no significant treatment-related effects on the kidneys, even at the indicated doses in rodents [185].

*Human studies*: Recent studies have shown that phthalates can affect kidney function in children, pregnant women, and adults [3,148,189,190,191,192]. Phthalates have a strong relationship with the urinary albumin creatinine ratio (ACR) and eGFR in children with CKD. Urinary phthalate levels of CKD children were lower than healthy children, possibly because children with CKD may have healthier eating habits [3,148,193]. A study in more than 400 women in Korea showed a significant positive correlation between phthalates and ACR in both single-pollutant models and multi-pollutant models [191]. In the co-exposure model, the phthalates have an additive effect on ACR. In a hypertensive population, phthalates were found to be positively correlated with ACR, β2-microglobulin, cystatin C, and negatively correlated with eGFR. Hypertensive people are more sensitive to early kidney injury due to phthalate exposure [4,194]. Compared with normal albuminuria patients, patients with microalbuminuria and macroalbuminuria had higher levels of phthalates urinary metabolites, which is unrelated to eGFR [195,196]. Over time, however, tubular damage from phthalates and increased oxidative stress follow a pattern that can affect kidney function due to long-term exposure, revealing an unexplored relationship between phthalates and the determinants of renal status [131].

### 5.3. Mechanisms of Phthalate Toxicity in the Kidney

Studies indicate that phthalates cause kidney disease through multiple mechanisms [192,197,198]. In human kidney embryonic cells (HEK-293T), phthalates were found to directly regulate mRNA translation, which was manifested as the inhibition of both cap-dependent and -independent mRNA translation in vivo [199]. In rodents, phthalates significantly increase oxidative damage and phosphorylated extracellular regulated protein kinases 1/2 (**ERK1/2**) expression [195]. Phthalate exposure also increased ROS, malondialdehyde (MDA) and DNA-protein cross-linked (**DPC**) levels and decreased GSH levels also in a dose-dependent manner [200]. An increase in protein levels of **NRF-2**, **HO-1**, and **GCLC** (responsible for GSH synthesis) was also observed. In addition, upregulation of **P53** and **BAX** proteins and the downregulation of **BCL-2** by phthalate exposure suggest that phthalate-induced apoptosis was activated by the mitochondrial pathway [201,202].

Phthalate exposure has also been shown to cause toxicity by promoting autophagy [187]. Oral administration of phthalates in pregnant rats increased **LC3II/I** protein expression, increased hedgehog interacting protein (*Hhip*) gene and protein expression, and inhibited hedgehog signaling, leading to autophagy in renal tubule cells and resulting in renal fibrosis [203]. In addition, maternal exposure to phthalates can activate the *Rhoa/Rock* pathway in the kidney of the offspring, inducing the occurrence of EMT, which may eventually lead to renal fibrosis in the offspring [204]. Peroxisome proliferator-activated receptors (**PPARγ**) significantly inhibited phthalate-mediated EMT induction [205]. Phthalate exposure reduced the protein expression of **PPARα** and **PPARγ** both in vivo and in vitro. Phthalates induce large accumulation of **AHR** and **AHR** nuclear transporter (*Arnt*) in the nucleus [206], exacerbating chronic kidney diseases.

These results suggest that phthalates can induce oxidative stress and apoptosis in the kidney through dermal, oral, and other exposure routes, ultimately leading to histopathological changes in the kidney. However, most studies have focused on rodents, and internal doses, which makes it difficult to assess the potential risk aspects of phthalates in humans.

## 6. Conclusions

**Figure 3:**
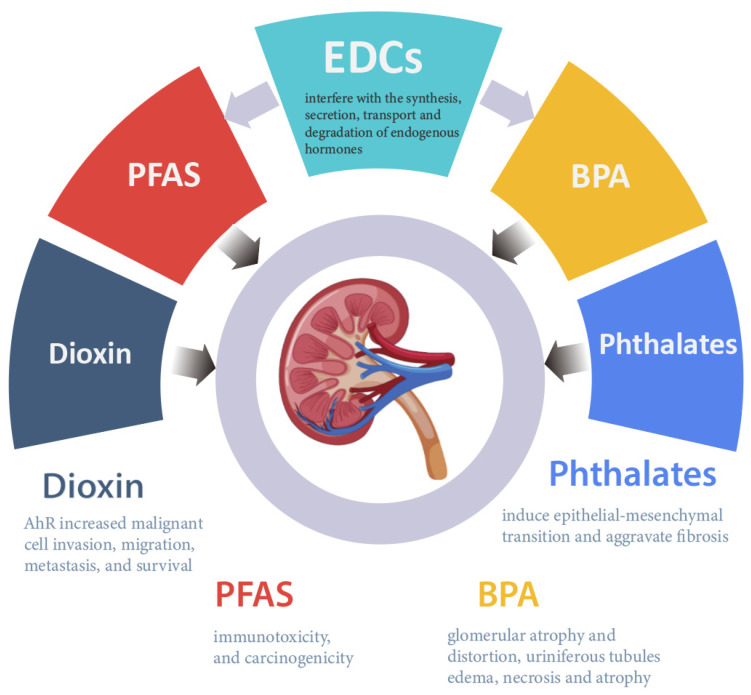
Diseases/conditions triggered due to exposure to EDCs.

This review summarizes and discusses the effects of EDCs on kidneys (Figure 3). EDCs cause proteinuria, affect eGFR, perturb the intracellular REDOX balance, and activate apoptosis and **AHR** pathways, eventually resulting in kidney injury and renal fibrosis. Most previous studies are l focused on the traditional environmental pollutants such as PCBs and BPA, whereas few studies focus on newer or emerging environmental pollutants. With the development and application of new materials, the types of environmental pollutants in water and food are constantly increasing, and new drugs and EDC substitutes are on the rise. It is clear that several gaps exist in the toxicity studies and the molecular mechanisms triggered by these pollutants. Mechanistic studies provide a sound basis for the identification of key molecular toxicity targets of EDC and provide theoretical basis for its health risk assessment. Hence, foundational knowledge on the mechanism of action of traditional EDCs on kidney is imperative for more effective prevention strategies and/or treatment options.

The toxicity of EDCs to organisms is not only caused by a single pollutant, but often comes from multiple pollutants. Therefore, future studies, could focus on chronic exposures from EDC mixtures at low concentrations.

Past work has shown that EDC isomers (example, dioxin) induce different biotoxicity. Hence, it is necessary to analyze major isomer components in human blood. Alternatively, the analysis of isomers can be used to distinguish the sources of different production modes and identify direct or indirect exposure routes in humans. Due to the differences in molecular structure, isomers may have different accumulation rates. Very few studies have focused on the composition of isomers in human blood and the potential harm to human body. Thus, these effects need to be addressed.

## Figures and Tables

**Table 1:** Typical EDCs and kidney diseases.

EDCs		Subjects	Effects
Dioxin	Animal Study	zebrafish	ballooning degeneration and necrosis of the renal tubules
		female aryl hydrocarbon receptor (**AHR**) knockout mice	inflammatory, acute tubular injury
		wild-type male mice	increase *CYP1A1* expression
		fetal mice	induce fetal hydronephrosis, hyperuricemia and fibrosis
		rats	increase serum creatinine and blood urea nitrogen levels, renal oxidative stress
	Human Study	CKD patients	polymorphic variants of *CYP1A1* overexpression
		human **AHR** mice	TCDD-induced hydronephrosis, altered **AHR** target genes
PFAS	Animal Study	dolphin renal epithelial cell	rapidly inhibit gap junctional intercellular communication
		Xenopus A6 renal epithelial cell	stimulate DNA/RNA, secondary protein structures, lipids, and fatty acids
		male and female rats	increase uridine diphosphate -glucuronosyltransferase (UDPGT) activity
	Human Study	human kidney cancer cell	increase *Acta2*, *Bcl2l1*, *Kdm1a* and *Kdm4c*, decreased DNA methylation
		human kidney cancer cell	upregulate *Nef2l2*, *Hes1*, *Ppara*, and *Ppard*
		CKD patients	decrease levels of hemoglobin, serum albumin, and eGFR
		healthy middle-aged women	increase the risk of diabetes
		RCC patients	increase RCC risk
Bisphenol A	Animal Study	rat kidney cancer cell	activate autophagy and apoptosis-related signaling pathways
		fish	glomerular atrophy, uriniferous tubule necrosis, severe hyperemia, and blood hemolysis
		mice	disrupts electrolyte balance,
		fetal mice	glomerular abnormalities and decreased glomerular formation
		rats	infiltrative and dilatative changes in renal tissues
		rats	increased lipid peroxidation and decreased levels of the antioxidants
	Human Study	human kidney cell	promotes kidney injury
		CKD patients	decrease in eGFR and renal function
		human fetus	lead to DNA methylation
Phthalates	Animal Study	rat renal cells	epithelial–mesenchymal transition and aggravate renal fibrosis
		fetal mice	induce EMT
		mice	significant perturbation of metabolic profiles and renal dysfunction
		mice	increase levels of nuclear factor erythroid 2–related factor 2 (**NRF-2**), heme oxygen-ase-1 (**HO-1**), and glutamate-cysteine ligase catalytic subunit (**GCLC**), induce apoptosis
		pregnant rats	lead to autophagy in renal tubule cells and result in renal fibrosis
		rodents	increase oxidative damage and extracellular regulated kinase ½ (**ERK1/2**) expression
	Human Study	CKD children	affect urinary albumin creatinine ratio (ACR) and eGFR
		Korea women	positive correlation between phthalates and ACR
		human kidney embryonic cells	inhibit of both cap-dependent and -independent mRNA translation

## Data Availability

Not applicable.
